# “… I would have left that man long time ago but, …” exploring circumstances of and motivators for repeat adolescent birth in Eastern Uganda

**DOI:** 10.1186/s13690-021-00662-9

**Published:** 2021-08-06

**Authors:** Dinah Amongin, Frank Kaharuza, Claudia Hanson, Annettee Nakimuli, Susan Mutesi, Lenka Benova, Lynn Atuyambe

**Affiliations:** 1 Department of Health Policy Planning and Management, Makerere University College of Health Sceinces, Kampala, Uganda; 2 Department of Obstetrics and Gynaecology, School of Medicine, Makerere University College of Health Sciences, Kampala, Uganda; 3 Department of Community Health and Behavioral Sciences, Makerere University School of Public Health, Kampala, Uganda; 4 Department of Global Public Health, Karolinska Institutet, Solna, Sweden; 5 Department of Disease Control, London School of Hygiene & Tropical Medicine, London, UK; 6 Department of Public Health, Institute of Tropical Medicine, Antwerp, Belgium; 7 Faculty of Epidemiology and Population Health, London School of Hygiene and Tropical Medicine, London, UK

**Keywords:** Adolescent, ‘Repeat birth’, Circumstances, Motivators, Coercion, Uganda

## Abstract

**Background:**

First birth before 18 years has declined in Uganda unlike *repeat adolescent birth* (=second or more births before age 20 years). We explored the circumstances of and motivators for repeat adolescent birth in Eastern Uganda.

**Methods:**

Between January and March 2020, we conducted a qualitative study involving 70 individual in-depth interviews with purposively selected respondents - 20-25-year-old women with and without repeat adolescent birth, their partners, and parents, in the communities of Teso sub-region. We conducted latent content analysis.

**Results:**

Four major themes emerged: poverty, vulnerability, domestic violence, and demotivators. Sub-themes identified under poverty were: “limited provisions”, “peasantry”, “large families”, “dropping out of school”, “alcohol abuse”, and “broken family structure”. Vulnerability included “marital entrapment” and “partner coercion”. Demotivators included: “abandonment”, “stern warning”, “objection to marriage”, and “empowerment”. Extreme poverty resulted in inadequate provision of basic needs leading to unprotected sexual activity in a bid to secure financial support. Following the first birth, more than three quarters of the women with repeat adolescent birth reported increased economic distress that forced them to remain in unwanted marriage/union, often characterized by partner coercion, despite wanting to delay that repeat birth. Women without repeat adolescent birth avoided a second birth by empowerment through: an economic activity, contraception use, and resumption of schooling.

**Conclusion:**

Repeat adolescent birth in Uganda is premised around attempts to address the economic distress precipitated by first birth. Many women want to delay that repeat birth but the challenges robbed them of their reproductive autonomy. Beyond efforts to prevent first birth, programs need to address economic empowerment, ensure contraceptive access, and school re-integration for adolescent mothers in order to prevent shortly-spaced repeat births.

## Plain English Summary

In the last 30 years, the proportion of adolescents (10–19 years) in Uganda having a first live birth before age 18 years has declined. However, among these, the proportion who proceed to have another live birth before age 20 years (=repeat adolescent birth) has not declined. We therefore set out to find out what motivated or were the circumstance under which these adolescent girls had repeat adolescent birth. We answered this question by interviewing people in Eastern Uganda: 1) women age 20–25 years who had first birth before age 18 years; those with and without history of repeat adolescent birth, 2) the parents with girls who had first birth before 18 years, 3) partners to women with repeat adolescent birth, and 4) community leaders.

We found that the financial challenges that had escalated following the first birth was responsible for the events or decisions that culminated into a repeat adolescent birth. In an attempt to find a solution to the financial challenges, the adolescent girls end up in unwanted unions with men and ultimately lost their power to decide on whether or not to get pregnant again. Many wanted to have this repeat birth later but were robbed of the opportunity to do so. The study findings suggest that beyond helping adolescent girls prevent the first birth, they need to be supported with economic empowerment activities, improved contraception services, and re-integration in school.

## Background

Adolescent fertility remains a major public health challenge in Low and Middle Income Countries (LMIC) that are home to over 95% of the global share of approximately 16 million births annually [1, 2]. Sub-Sahara Africa alone accounts for over 23% of this figure. Whereas there has been some progress in reducing adolescent pregnancy, variations in achievement exist between and within countries [2–7] including, in the three major East African countries- Tanzania, Kenya and Uganda [8, 9]. In Uganda, the age specific fertility rate among girls 15–19 years declined from 195 births per 1000 women per year in the 1990 Uganda Demographic and Health Survey (UDHS) to 132 births per 1000 women at the 2016 UDHS [10]. In the past 30 years, the percentage of Uganda women age 20–24 years who experience first birth < 18 years of age declined from 41.7% in 1988/89 to 28.4% in 2016. However, the proportion of these women who proceed to have *repeat adolescent birth* (=a second or higher order live birth < 20 years following first birth < 18 years) [11] did not decline over the three decades; remaining at over 50%.

In order to curb down births before 18 years of age, Sub-Sahara Africa countries such as Uganda instituted laws against early marriage and enacted the defilement act [12, 13]. However, the implementation of these laws is hampered by the cultural norms that promote early marriage in exchange for bride price [14–17] that is often used to meet financial obligations at the girl’s birth family [18]. Further, pursuit of the legal course of action following defilement or pregnancy of a girl below 18 years of age is usually compromised in Uganda; families settle matters out of court and levy a ‘fine’ on the man [19, 20]. This ‘fine’ is not included in the bride price settlement. In the event that a woman opts to leave the marriage, for whatever reason or duration of the marriage, her birth family is expected to return the bride price in full [21]. However, in a bid to reduce violence against women, this cultural practice of bride price refund was outlawed by the Uganda court of law in 2018 [17, 22–25] but, the extent of dissemination or implementation of this ruling is still limited. This marriage is usually traditional (customary) although some of the girls cohabit with the partners.

Adolescent births commonly occur in settings of: high poverty, low educational attainment, and early marriage [1, 3, 15, 26–28]; thereby affirming previous research findings that these double as outcomes of the adolescent births [29–31]. First adolescent births are mostly unplanned with many of the adolescent mothers reporting having not expected to get pregnant [1, 32, 33] while others are wanted [34, 35].

Evidence from higher income settings show that following first birth, some of the adolescent women with repeat birth decided to complete their family size while others, were coerced to have a repeat birth by the partner or lacked parental support and vocational opportunities [34, 36, 37]. These studies reported that women who set personal goals and aspirations avoided the repeat pregnancy. We were not able to retrieve qualitative studies in sub-Sahara Africa that explored the reasons or circumstances for repeat adolescent pregnancy/birth [38] and yet, repeat adolescent birth forms a substantial proportion of all births < 20 years of age [11, 39]. Further, subsequent live birth in adolescence may compound on the negative outcomes of adolescent childbearing [1, 27]. We explored the circumstances of, and motivators for, repeat adolescent birth (=second or higher order birth < 20 years of age following first birth < 18 years of age) in Eastern Uganda in order to inform programming.

## Methods

### Study design and context

We employed a qualitative study design using in-depth interviews (IDIs) among respondents in the communities and locations of Soroti and Katakwi districts in Teso sub-region, Eastern Uganda. The in-depth interviews were chosen because we needed to obtain personal experiences regarding this very sensitive topic - adolescent birth [40]. Further, for this study, we used the Standards for Reporting Qualitative Research (SRQR) reporting guidelines for qualitative studies [41].

The districts of Soroti and Katakwi are inhabited by the Iteso – an ethnically homogenous Nilo-Hamite population. Soroti district was selected on account of being the commercial hub and Katakwi represented the other districts in the region. Soroti district is the central hub for Teso sub-region. Katakwi represents the other districts. Soroti district has 10 sub-counties and 26 parishes, Katakwi has 9 sub-counties and 46 parishes. According to the Uganda National Housing and Population Census (NHPC) Survey in 2014, most of the people reside in rural areas; 247,187 (83.3%) in Soroti and 156,943 (94.4%) in Katakwi. Of the total population in these districts, 92,761(55.8%) in Katakwi and 163,542 (55.1%) in Soroti were aged less than 18 years. In 2016, Teso sub-region had the highest childbearing rates with 31.4% of the adolescent girls 15–19 years having started childbearing, compared to the national average of 24.8% [10].

### Study participants and procedures

In the two districts, we employed purposive sampling to select the participating sub-counties and subsequently four parishes with most adolescent pregnancies in each of the participating sub-counties based on guidance provided by the Assistant District Health Officer, Maternal Child Health in each district. In Soroti district, we purposively recruited respondents from Katine, Gweri, Arapai and Asuret sub-counties for the rural category and from Eastern, Municipal and Northern Divisions for the urban category. In Katakwi respondents were from: Usuk, Magoro, Toroma and Kapujan sub-counties for the rural category and, Central Division for the urban category.

Study participants were: 1) women age 20–25 years with or without repeat adolescent birth following first birth < 18 years of age, 2) spouses/partners of women with repeat adolescent birth, and 3) parents of women with or without repeat adolescent birth. The parents and partners were largely unrelated to the women interviewed. We recruited them from their homes/communities using purposive sampling. To identify target respondents, the Assistant District Health Office introduced us to the sub-county village health teams (VHT) coordinator who subsequently linked us to the parish coordinator. Within the parishes, the VHT coordinator mobilized other VHT members to work with us. We briefed the VHT members on the selection criteria which they used to identify potential respondents. Being level I of the health care system and knowledgeable about their village community members, we chose village health teams (VHTs) led by the sub-county coordinator, as the contact point to identify and recruit target respondents. The study team confirmed eligibility of the potential respondents and ensured informed written consent was sought.

Interview guides capturing participants’ socio-demographic characteristics as well as interrogating the circumstances informing first and repeat/non-repeat adolescent birth were developed, pre-tested and accordingly adjusted with input from non-participating communities in Soroti (Table 1). These were translated into Ateso by two bilingual research assistants. To ensure consistency of information, these guides were back translated into English by an independent bilingual social scientist with research experience.

**Table 1:** Main interview questions for the study “Circumstances of and motivators for repeat adolescent birth in Eastern Uganda”

**Main questions asked regarding repeat adolescent birth** (Category of respondents were: women with repeat adolescent birth, partners, and parents to daughters with repeat adolescent birth)	
1. Please share with us the circumstances under which you/your partner/your daughter had the first childbirth? *Probe: About upbringing, schooling, employment, marriage/union, living alone, age at conception and childbirth, contraception use/non-use, living alone, etc)*	
2. After the first pregnancy was confirmed until the repeat adolescent pregnancy and birth, please tell us about what happened to you/your partner/your daughter. Take us through the events. *Probe: for marriage/union, who she lived with, where she gave birth, who was caring for her, contraception use/non-use, schooling, employment/work, etc* *Partner: inquire if the first and repeat childbirths from this lady are all his. If only the repeat birth, explain the circumstances and motivators for it.	
3. Help us understand, from your perspective, what influenced you/your partner/your daughter to have another child before age 20 years? *Probe for: Economic, social, personal desire, marriage related, family support, peer influence, community, and reproductive health services related factors.*	
4. At the time you/your partner/your daughter had another birth, was this the preferred time? Please explain your response.	
5. Of all the things you have shared with us, which was the biggest influencer for you/your partner/your daughter to have another child before age 20 years? *Probe for reasons/influencers. Summarize the reasons eg 3.*	
**Main questions asked regarding no-repeat adolescent birth** (Category of respondents were: women with no-repeat adolescent birth and parents to daughters with no-repeat adolescent birth)	
1. Please share with us the circumstances under which you/your daughter had the first childbirth? *Probe: About upbringing, schooling, employment, marriage/union, living alone, age at conception and childbirth, contraception use/non-use, living alone, etc*	
2. After the first pregnancy was confirmed until the age of 20 years, please tell us about what happened to you/your daughter. Take us through the events. *Probe: for marriage/union, who she lived with, where she gave birth, who was caring for her, schooling, contraception use/non-use, employment/work, etc*	
3. Help us understand, from your perspective, what influenced you/your daughter not to have another child before age 20 years? *Probe for: Economic, social, personal desire, marriage related, family support, peer influence, community, and reproductive health services related factors.*	

We collected the data between 06th January 2020 and 20th March 2020. The principal investigator (DA) together with four trained research assistants fluent in both English and Ateso conducted and audio recorded all the interviews in safe and convenient spaces for the respondents to the point data saturation was attained – no further new answers to the questions received. The interviews lasted approximately 1 h following completion of the informed consent processes.

### Data management and analysis

Interview recordings were transcribed and translated verbatim Ateso into English ensuring no alteration in meaning. Three of the authors (DA, SM, and LA) read the transcripts several times while agreeing on areas that needed more clarity. We conducted Latent content analysis was conducted using the approach by Graneheim and Lundman [42]. We embarked on analyzing the text in each transcript and interpreted the underlying message. We generated codes using framework analysis which we then grouped data into sub-themes and themes. Emerging themes were explored in subsequent interviews until thematic saturation was reached. For the demographic characteristics, the mean age of the women at first birth was expressed as a mean whereas other parameters were left as total numbers.

#### Quality control

Data collection was conducted by bilingual trained experienced research assistants. These data collectors transcribed interviews together with the principal investigator. Each proof-read their transcripts. For purposes of checking concordance, we sampled out six audio recordings from women with and without repeat adolescent birth (3 from each), had two people independently transcribe them and compared the transcripts.

## Results

### Background characteristics of respondents

We conducted a total of 70 interviews; 28 women with repeat adolescent birth, 15 women without repeat adolescent birth (=one birth before 18 years and no other before 20 years), 9 partners of women with repeat adolescent birth, and 18 parents to women with and without repeat adolescent birth (11 and 7, respectively) (Table 2).

**Table 2:** Demographic characteristics of the respondents for IDIs in Teso sub-region Uganda

Category of respondents	Women with repeat adolescent birth (***N*** = 28)		Women without repeat adolescent birth (***N*** = 15)		Partners to women with repeat adolescent birth (***N*** = 09; 5 urban)	Parents to women with repeat birth (***N*** = 11; 5 rural)	Parents to women without repeat birth (***N*** = 7; 4 rural)
	Urban (10)	Rural (18)	Urban (*n* = 7)	Rural (*n* = 8)			
Mean age at first birth (years)	16.0	15.9	16.6	16.8	NA	NA	NA
**Education**							
No/incomplete primary	7	13	2	3	4	9	2
Complete primary	2	4	0	2	1	0	2
Secondary and above	1	1	5	3	4	2	3
**Earning cash at interview**							
yes	4	2	6	3	7	2	4
no	6	16	1	5	0	9	3
**Father to both 1st and repeat birth**					4		

*NA* Not applicable

Women with repeat adolescent birth had mean age at first birth of 16.0 years (range, 13–17 years) compared to 16.7 years (range, 15–17 years) among women without repeat adolescent birth. This pattern was similar by rural-urban disaggregation. Those with repeat adolescent birth had lower education compared to those without repeat adolescent birth, irrespective of residence; only two out of the 28 had attained secondary education compared to eight out of the 15 (more than half) of the women without repeat adolescent birth. Further, more women without repeat adolescent birth earned cash income compared to those with repeat adolescent birth; nine out of the 15 compared to six out of the 28. More women in the urban residence earned money compared to those from rural residence, irrespective of adolescent birth history. Most of the parents, 12 out of 18, to the women interviewed were subsistence farmers with no direct cash income. Most parents who had cash income, however meagre, had daughters who did not have a repeat adolescent birth. The mean age of the partners interviewed was 26.0 years (range, 22–42 years) and four out of the nine fathered both the child from the first adolescent birth and the second (repeat) adolescent birth.

#### Context of female adolescents’ lives

Many adolescent respondents were raised in vulnerable environments characterized by extreme household poverty, alcoholic parents and broken family structures due to death, separation of parents, or simply a child being raised by other relatives. Broken family structures were common among the rural residents. As a result, many adolescents were exposed to financial distress and domestic violence.

We determined four themes explaining the circumstances of and motivators for repeat adolescent birth. Among women with repeat adolescent birth, the partners, and parents to the women with repeat adolescent birth, we determined three themes; poverty, vulnerability, and domestic violence (Table 3). Among women without repeat adolescent birth and parents to women without repeat adolescent births, we determined one theme; demotivators for repeat adolescent birth.

**Table 3:** Example of the abstraction process for the study on repeat adolescent birth in Eastern Uganda

Themes	Sub-theme	Sub-sub-theme	Exemplar quotes
Poverty	Limited provisions		“… my parents cannot pay school fees for all the 7 children so it is only one boy who is in school now …”
			“I had no knickers and when I could go into periods, I would have nothing to use and that could trouble me a lot …”
	Large families		“… we are 8 children, that number was big...So it was too much and yet there was no money …”
	Dropping out of school		“I stopped going to school when the pregnancy was 2 months; I could no longer concentrate in class because I was only thinking of the pregnancy …”
	Alcohol abuse		“… whenever he [father] would get money, he would go drinking and leave us … he was not bothered …”
	Broken family structure		“When my mother left, there was no one to pay my school fees …”
			“When my father died, my mother could not raise my school fees …”
Vulnerability	Marital entrapment		“… others are just forced to get married …”
			“… I cannot go back and burden my family.”
			“I decided to get married to a person who could provide them [family] with food and he is paying school fees for my sisters up to now …”
	Partner coercion		“… he [partner] could not allow me use any family planning method even up to now, he does not want me to use it; he is very against it and very tough when it comes to using family planning …”
			“… he [partner] touched my arm and felt it and he ordered me to remove it immediately; he was very bitter.”
Domestic violence	Abuse		“… he [the father] would come back at night and start abusing us, chasing us out of home …”
			“The condition at home was also hard; the kind of life we were living in was not good …”
Demotivators for repeat adolescent birth	Partner abandonment		“Unfortunately, he [partner] no longer is helping me … I am struggling on my own …”
			“… he refused me completely … actually I have gone through a lot and it has made me stay without getting another child.”
	Stern warning		“I shouted at them to leave issues of my child alone so I became tough on her …”
	Objection to marriage		“No, no, we have never wished any of them to get married, we instead encouraged them to study …”
			“I did not marry; I stayed home with my parents.”
	Empowerment	Economic activity	“My mother gave me capital to start business …”
		Contraception use	“Am now using injectaplan for 3 months …”
		Resumption of schooling	“I had to suggest to her to do this course she was positive so I took her.”

### Poverty

#### Limited provisions

Extreme household poverty was an overarching situation that was narrated as accounting for the decisions that resulted in the first and subsequently, the repeat adolescent birth; the women reported that they were compelled to take on male sexual partners for financial support in order to bridge this gap. This was among both rural and urban residents. Almost all the women narrated that their families were not able to adequately provide basic needs such as scholastic materials, and other essentials like underwear and menstrual pads. They said that the parents often struggled to raise school fees and often prioritized the boys.

*“… being a girl there are some necessities that you may be lacking and … I thought that I also want to get some partner of mine who will also help me and buy for me also some things like that …”* (Rural woman with repeat adolescent birth)

The limited provisions were aggravated by; peasantry family settings, large families, alcohol abuse by parents, and broken family structures.

#### Peasantry (subsistence farming)

Women with repeat adolescent birth and their parents reported that they were mainly subsistence farmers with meagre incomes that were inadequate to meet all the provisions for their families. This was common even among the urban residents.

#### Large families

*Eight of the twenty-eight* respondents originated from families having eight or more children. The large number of children and dependents made it difficult for the parents to provide basic needs to their daughters. Most of the parents also concurred that the large families made it difficult for them to provide everything for the children. Heavy dependency was more evident among the urban residents.

*Alcohol abuse by parents*: In the rural areas, high alcohol consumption by especially fathers, was reported to have aggravated the economic deprivation of the families. Many of the women from rural residence reported that their parents expended most of their resources on alcohol.

*“… there was no one to pay my school fees, my father would not bother giving me but he would go to drink whenever he would get money so I had to leave school and get married.”.* (Rural woman with repeat adolescent birth)

#### Broken family structure

Twelve of the eighteen women with repeat adolescent births from rural residence had a history of parental separation, death of one parent, or simply being made to live with other relatives; they could not adequately provide for them. Dropping out of school due to lack of school fees or materials was a common narrative. In attempts to address their vulnerability, they opted to get into relationships with men resulting in the first birth and repeat adolescent birth(s). They hoped that marriage would enable them escape from these deprived environments.

#### First pregnancy and dropping out of school

Nearly all the women from both rural and urban areas reported disrupted school attendance. They said that first pregnancy occurred while they were still in primary school although, more than half said that they had already lost interest in school because of the challenges in fees, scholastic materials, and menstrual hygiene products. -

*“… when it comes to paying school fees, it was never paid on termly basis. You would study from first term to third term and when it comes to the time for paying school fees you would be told that there is no money!”* (Rural woman with repeat adolescent birth)

Following the first birth, all the women with repeat adolescent birth (even the few who were not in union) reported that they did not resume education as their parents cited lack of money. Nearly all the women from the rural residence reported that they opted to get into union primarily seeking economic relief.

On the other hand, while acknowledging their limited resources, the majority of parents and guardians categorically stated that they provided most, if not all, the basic needs of their daughters. The fathers stated that the first birth was perhaps a mistake but the repeat births were a result of bad behavior, promiscuity or desire for marriage on the part of the girls.

*“she was promiscuous and that was what was driving her!”* (Rural father of a woman with repeat adolescent birth)

While another urban residence father thought it could possibly be the stupidity of children.

*“I don’t know whether it is the stupidity of children! My daughter told me the boy used to give her food items. Just those food items! … [*takes a long pause*]...”*

On the mismatch between the statements of the parents and the girls regarding the provision of basic needs, the mothers however acknowledged that there was usually no discussion with the girls about their needs. The father, who usually controlled all the resources at home, unilaterally determined ‘what and when’ to give them for their basic needs.

### Vulnerability

Women with repeat adolescent birth reported multiple vulnerabilities including marital entrapment and partner coercion that culminated in the repeat birth.

#### Marital entrapment

At the point of first pregnancy of a minor, almost all the women and the parents reported that, families usually settled for dialogue involving payment of a fine in the form of cows and money by the perpetuator instead of prosecuting them for defilement, as dictated by the law. The law enforcers reportedly encouraged families to negotiate and settle matters out of court. Parents who had tried to take the legal action were often frustrated by the police corruption. Most of the women from both residences reported being forced to go and live with the man during or shortly after the payment negotiations, in a transfer of financial responsibility for the pregnancy. The women stated that their birth families did not want them at home with the pregnancy; they were considered a burden.

*“… they said ‘there is no problem she will first remain there, even if she dies, we shall also come and do what we shall do’. So, I then had to remain living there … I still had to stay because I didn’t want to take the burden to my people …” .* (Rural woman with repeat adolescent birth)

However, four out of the five rural and two out of the six urban parents interviewed (total six out of eleven) stated that they did not force the girls into marriage but only wanted the man responsible cater for their daughter during the pregnancy and pay a ‘fine’ to compensate for their investment in their daughter. Despite the parents saying this, the women with repeat adolescent birth stated that their parents pursued dowry payment following the first birth. Nearly all of these women said they had to remain in the union due to lack of an alternative and they feared that the dissolution of the marriage would burden their families to return the dowry. Once in a union and under the expectation to bear more children, the women found themselves unable to prevent the repeat adolescent birth. As a woman from the rural residence stated,

*“… you know as someone has married you, so you cannot go and just stay in someone’s place like that! Someone will want what? (pause) Children!”*

Most of the women with repeat adolescent birth stated that they personally decided to have another child as either a means of: securing their relationship with the partner (new partner or the one responsible for both first and repeat birth), increasing her family size or, simply fulfilling the expectation of producing more children. This was more common among the rural residents. As stated by a woman from rural residence, she thought a second birth would help stabilize their relationship,

*“… actually, what I thought was that when I get another child, he would now settle down and that is what drove me to get a second child … I thought by doing that, he will be convinced and he opens up his mind to know that am now ready to be a wife and he stays with me but* [pause]...*but I realized that men are not trusted and they will never talk the truth …”*

Another woman from rural residence said it was good to have your children as they are a source of help,

*“… it is also good for you to have your children. Sometimes, they help you … like now that elder one you can send her that go and do for me something like this like that … .”*

The ability to channel financial resources from the relationship to support their birth families also motivated longer union tenures and therefore the repeat pregnancy, as reported by eight out of the 17 women with a history of single parent (especially female-headed) families. For the women from urban settings, the support was mainly to ensure continued schooling for their siblings whereas those in rural areas aimed at getting bride-price paid; this would be used for support. This selflessness was a motivator to stay in the union and ultimately the repeat pregnancy. As a woman- a half orphan- from an urban area narrated,

*“..I feel bad but I have nothing to do because I want to see my sisters push with their studies otherwise I would have left that man long time ago but I look at my sisters and wonder how they can survive; we are orphans and our mother is not able to support them so I have to stand in the gap because there is none who can be there for them … I had to push myself into marriage”.*

Whereas another orphan from the rural area narrated,

*“… so I thought that it was better for me to go for marriage and I see how to help my brothers on the side of cows maybe they would purchase some piece of land somewhere where they would also settle.”*

Among women from rural residence, seven out of the 18 reported being influenced by peers who had already given birth to get into relationships and subsequently bear children. In the urban area, peer influence to get into union as a solution to the poverty and violence was at the point of first pregnancy and following this, their peers began avoiding them.

*“… .my friends would also tell me to get a boyfriend … .I wanted to stop suffering..”* (Rural woman with repeated adolescent birth)

These women reported that their peers and older women in the community put them under pressure to have the first birth and following this, to have more births as soon as possible in order to have time to concentrate on raising their children. The fear of failure to attract other suitors was used to compel them to accept unwelcome partners or to cement the relationship by having more children. As an example, a woman with repeat adolescent birth from rural residence stated that, following abandonment by the first partner, her own mother encouraged her to stop contraception and get pregnant for the second partner to accelerate the decision on marriage. She said,

*“… She said I should stop so that I can conceive because I cannot stay with the man when I don’t have a child; that it is a child that makes a man to love you!”*.

Unfortunately, this man also abandoned her as soon as she had the repeat adolescent birth.

Regarding the partners interviewed, four out of the seven stated that they were already in a union and more children were expected out of the union. They however acknowledged the value of spaced childbirth and were considering starting a contraceptive method. Two partners said they had already encouraged the woman to use contraception.

#### Partner coercion

Nine out of twenty eight of the women in the rural and urban areas reported that the fathers of the first birth forced them to have the repeat adolescent birth. Most of these women had opted to use contraception following first birth but were coerced by the partners (some by physical violence) to stop contraception and conceive again. The partners threatened to end the relationship and financial support. As a woman in the urban area state,

*“… and then he got me when I had put the injection [*points at implant site in arm] *for family planning and he then told me that we should remove that injection and when I refused, he started to beat me and took me to the hospital and removed it!”*

Some new partners, demanded a child as a condition for continued financial support and perhaps marriage. These women reported that much as they did not want a second child soon, they did not want to risk losing this opportunity. As a woman in the urban area stated,

*“… he started quarreling that am not giving him a child, it seems am barren and that is how I got pregnant but I did not intend to get a second child because my first child was still not well …”*

For two women, the repeat birth was a result of rape by men they were acquainted with. One got married to the perpetrator and opted to have more births. The other reported that the perpetrator ridiculed her as a prostitute – due to first birth. None of these women sought legal redress because they perceived that no one would believe them since they had already given birth.

### Domestic violence

Many rural residence women (9 out of 18) opted for marriage and ultimately, more childbearing, as a means of escape from the domestic violence at their birth home in forms including: deprivation of food, physical abuse, verbal abuse, and excessive manual labor/child abuse from their parents or guardians. They said that they could not even think of returning into the abusive homes. Women from broken family structures faced more violence compared to those who were living with both parents. As a rural woman who was orphaned reports,

*“… the words when you are in someone else’s home someone who is different and not your mother, the person favors giving you harsh words … there is so much abusing like that to the extent that my aunty told me that ‘you people are like dogs. It wasn’t me who told your mother to pass on’ …” .*

Among the women from rural residence with parents having extreme alcohol consumption, violence got worse when the parents were drunk. As one woman from rural residence narrated;

*“… When he [*the father*] is sober the stay would be good but the moment he drinks, things would change; he would start barking and chasing us...”*

### Demotivators for repeat adolescent birth

From the interviews with women without repeat adolescent birth and parents to women without repeat adolescent births, we identified four demotivators for repeat adolescent birth: partner abandonment, stern warning, objection to marriage, and empowerment. A common feature was the endurance of more hardships following the birth than prior to the pregnancy. As an example, a woman from rural residence stated,

*“… I have gone through a lot and it has made me stay without getting another child; problems that I have experienced right from the time I got pregnant... I vow not to repeat the same mistake I did!*”.

#### Partner abandonment

Nearly all of the women, irrespective of the residence, reported abandonment by the man as soon as the pregnancy was pronounced. The women and parents reported that the men who had initially been deceiving them with ‘financial support to bridge the gap in their basic needs’ suddenly abandoned them by either denying the pregnancy or escaping from the area. Some returned later and accepted responsibility for the pregnancy. The few women who had been sent off to be with the man until they gave birth returned back to their birth families due to physical violence from the men. The men were reported not to cater for the children either and in situations where they tried, it followed use of force/threats, was sporadic, and meagre. The women reported that this lack of partner support for the child put a lot of emotional and financial strain on them because their own families, on most occasions, could not afford to meet the child’s needs either. The women echoed that they had come to the realization that their future had been ruined and these men; they had nothing to offer them. As an urban woman stated,

*“… No he doesn’t help me at all! Not even a call to find out how the child is doing. Nothing!...”.*

#### Stern warning and counselling from parents

The women from both rural and urban residence, stated that their parents expressed great disappointment in them and gave them stern warnings not to get pregnant again while still at home. The women reported that this reaction was a source of great emotional and psychological trauma for them. This was exacerbated by negative social labelling and feedback from the community members; that they were spoilt girls who would ruin others. Almost all the parents of women without repeat adolescent birth, irrespective of residence, stated that they sternly warned and counselled their daughters against having another birth. As one father from the urban residence stated,

‘ … *I told her … for her to have another child in my home was not acceptable! Not when she is still with me!...*”.

Eight of the fifteen the women without repeat adolescent birth reported that their parents had initially chased them out of home and only allowed back on condition they did not conceive again. In the interim, they had to live with either the man responsible or his relatives. This category of stern parents went on to support the girls with either economic activities or schooling while catering for the child.

#### Objection to union/marriage

All except two women without repeat adolescent birth reported that they were not in a union nor had their families coerced them into it. Those who had initially been sent to be with the partner returned shortly after giving birth. They said that their parents gave them a chance to opt out. A few women reported that their parents actually rejected the option of marriage, citing their young age, contrary to the advice from other community members.

#### Empowerment

The above challenges and reactions shaped the strong personal resolve by women to prevent a repeat adolescent birth with the following empowerment actions taken in isolation or in combination, irrespective of residence: 1) empowerment of the woman through an economic activity, 2) contraception use, and 3) school resumption.

##### An economic activity

Nine of the fifteen women without repeat adolescent births, irrespective of residence, reported getting start-up capital from either parent shortly after giving birth; they started an economic activity. The parents reported that they opted to do this to reduce girls’ dependency on men for financial support. The activities were such as: setting up a road-side stall to sell tomatoes, making cassava chips, and brewing alcohol, etc. These activities, the women said, though relatively low yielding (generating 5000 shillings/ two United States Dollars daily), delivered significant financial independence and insulation against the demand for sexual favors in exchange for very little money. As an urban woman acknowledged support from her mother,

*“… I then started to fry chips* [cassava] … *My mother first gave me 10,000/=* [approximately 3 United States Dollars]*, then with that 10,000/= I bought cassava for 5,000/= and then I bought cooking oil two half liters. And then I realized that it started adding up until my money increased … and then some I also would take it to the savings group.”*

##### Contraception use

Majority of the women without repeat adolescent (10 out of 15) reported that they used modern contraceptive methods to deliberately delay another pregnancy within an active sexual lifestyle, that continued even while living with their parents. They acknowledged that it was not easy to completely avoid having sex. This was most common among women engaged in their own economic activities. Although some parents reported that they suspected that their daughters were using contraception, it was not discussed with them. They were happy that their daughters had managed to find a solution.

The women without repeat adolescent birth also reported awareness of men’s use of deception to acquire sexual favours without carrying responsibility for the pregnancy and subsequent child support. Some, mainly urban-based women, supported by a few parents, opted to retain control of the situation even in scenarios where a partner was against contraception.

##### School resumption/continuation

In almost all the scenarios of adolescent pregnancy by learners, the schools immediately expelled the girls. A few of the women reported that their parents excused their having given birth and promptly supported resumption of schooling or acquisition of technical skills (most commonly tailoring and hairdressing) from vocational institutions. They resumed school shortly after giving birth. In these instances, the families took charge of looking after the infant. These opportunities were most common in the urban settings. As a father from the urban area narrated,

*“… I wanted my daughter to go back to school … So we thought it would not be good to simply leave her, so we took her for a tailoring course.”.*

## Discussion

In this paper, we explored the circumstances and motivators for repeat adolescent birth in Soroti and Katakwi districts in Teso sub-region, Eastern Uganda. Individual in-depth interviews were conducted among: women age 20–25 years with and without repeat adolescent birth, parents to women with and without repeat adolescent birth, and partners to women with repeat adolescent birth. We found that repeat adolescent birth was largely driven by attempts, among both rural and urban adolescent women, to escape from family-related socio-economic challenges through developing financially supportive sexual relationships and marriages. However, the hardships which were present at first pregnancy, for many, only got worse following first birth. Below we discuss four key findings.

First, rampant household poverty compromised the provision of basic needs for the girls and led to interrupted school attendance. This situation was compounded by the large family sizes, parental alcohol abuse, broken families, and domestic violence. Largely, the adolescents had first pregnancy while still at school and dropped out once confirmed pregnant. Previous quantitative studies in Uganda and other LMICs affirmed that adolescent first pregnancy and birth, occurred mainly in situations of: economic deprivation, broken family structures, domestic violence, and cultures that promote early marriage and bride-price payment [1, 2, 12, 43]. These were also the situations where schooling was not guaranteed for the girl child [28, 43] including low continuation following pregnancy [31, 44] - as was found in our study. Our finding of poverty as central to the first birth and subsequently repeat adolescent birth agrees with the previous research findings that, early adolescent birth was driven by sexual exploitation of the girls in return for material incentives – transactional relationships [45–49]. Families see girls, as a source of revenue (=bride-price) and therefore marry them off at the earliest excuse irrespective of the legislation against early marriage [12, 43]. Enforcement of the defilement law in Uganda is weak with the collusion of law enforcers keen to settle matters at family level [20]. As seen in our study, parents often sent the girls off into marriage/union at first pregnancy and used whatever tactic possible to get bride-price – material/financial compensation, irrespective of the legislation. It is therefore important that household poverty is tackled and adolescent mother empowered through economic activities, as key steps in decreasing the vulnerability of adolescent girls to sexual exploitation.

Second, majority of the respondents reported being trapped in marriage/union and ultimately further childbearing in an effort to: get financial support, secure the relationship, meet the demands of the spouse or new partner, and meet the cultural expectations of bearing more children, despite preferring to delay the second order birth. For those who were rejected by the father of the first child, they had to get another partner shortly after giving birth. Therefore, while the first birth was described as unintended, the following pregnancy was largely intended, albeit not always. Along with this was partner coercion including physical violence in case the woman tried to avoid a repeat adolescent birth using contraception. Once married, adolescent girls get trapped; they lose individual power over their fertility decisions and resign into further childbirths. It can therefore be argued that marriage diminishes the power adolescent girls could have had if they had not been married; their agency is taken over by the men. The younger the adolescent girl, the more vulnerable she is due to for example, low economic empowerment and developing decision-making ability [50]. Patriarchy and coercion thus become the controlling element of their lives [48, 51, 52]. Our finding of partner coercion over contraception use and general low contraception adoption by adolescent mothers, aligns with previous findings of low uptake among adolescents, including in Uganda [53, 54]. Several reasons, including partner refusal have already been cited as one of the factors responsible for low uptake among adolescent women in Uganda [10]. As evidenced by our findings, the women wanted to delay the second birth but had no viable alternative other than to: get another man for financial support, get married, or simply resign to further childbearing. Uganda women’s desire to delay the second order birth has been reported in another study that we conducted using previous UDHS data among women age 20–24 years [11]. This calls for enforcement of the legislation against early marriage and that, the underlying causes, such as poverty, are tackled. Further, contraception should be provided in a manner that promotes a woman’s autonomy.

Third, adolescent girls who were empowered through: starting an economic activity, contraception use, and continuing with schooling or acquisition of vocational skills, did not have a repeat adolescent birth. These girls had resolved to avoid the repeat adolescent birth for reasons including: abandonment by the partner, stern warning with counselling from parents and objection to marriage. These results align with previous findings that repeat adolescent pregnancy can be delayed or averted by provision of viable alternatives such as: school continuation, economic activity, parental support, and contraception use [36, 55]. This calls for renewed efforts to ensure school continuation, provision of contraception to adolescent girls that ensures autonomy, and economic empowerment of adolescent girls.

Lastly, a few women made personal pragmatic decisions to bear more children. This finding that some of the women made individual decisions to have the repeat birth for reasons, such as a desire to have more children, aligns with previous findings that not all the repeat births are unwanted, but rather, adolescent girls make pragmatic decisions whether to or not to have another pregnancy/birth [34, 37]. These two qualitative studies, conducted in the United States of America, explored repeat pregnancies/birth in adolescence. Conroy et al. [37] explored perceptions of teen mothers towards their own decisions or behaviors that may have played a role in preventing or averting a repeat pregnancy within the first year after the first birth and found that, those who did not have repeat pregnancy intentionally avoided the pregnancy and choose to use contraception. Reddy et al. [34], explored the interpretations of motherhood and the life circumstances of multiparous mothers with rapid repeat births. Repeat births were seen as a method of building families, reinforced their adult motherhood identity, and acted as a stabilizing factor for relationships. These results defer from ours with regard to the major narrative; economic deprivation as a push factor into sexual relationships or marriage, in a bid to get financial or material support. The results highlight differences in economic opportunities and the cultural norms between the settings. This suggests that whereas interventions in the LMIC setting should address economic deprivation and domestic violence in order to decrease vulnerability of adolescent girls to sexual exploitation, there is a segment of adolescent mothers who may make pragmatic decisions to have a repeat adolescent birth. They need support to fulfil their reproductive intentions.

### Strength and limitations

This study was the first in Uganda, a LMIC, to explain repeat adolescent birth. We triangulated findings from different respondents - parents to the women and partners - beyond the women themselves unlike previous qualitative studies that interviewed only the women. This gave a more comprehensive view of the motivators and circumstances for repeat adolescent births. Further, we conducted our study using a community sample of rural and urban residents unlike other qualitative studies that used an institutionally drawn sample that potentially introduced a challenge regarding transferability. We do acknowledge that this study involved recall of information and the time lapse may have affected precise recollection of all events.

Additionally, the sensitive nature of the topic might have inclined respondents to provide socially desirable responses. During the interviews, four women with repeat adolescent birth broke down while narrating how the births happened. The interviewers allowed them to recompose and only proceeded when they signaled that they were comfortable to continue with the interview. They declined our offer to provide a counsellor, confirming their desire to share their testimonies to help other girls.

Nonetheless, this initial inquiry is a major step towards understanding repeat adolescent birth following first birth < 18 years of age in a LMIC.

The principal investigator, DA, is a medical doctor with specialized training in Obstetrics/Gynaecology and Public Health. Having worked as a clinician, health program implementer, and reproductive health advisor to the Ministry of Health, this technical background may have affected the interpretation of findings. To safeguard against this, qualitative inquiry was conducted with a multidisciplinary team that included social scientists; LA & SM.

## Conclusion

Our findings suggest that escalation of economic distress following first birth was the overarching reason for repeat adolescent births. Along with domestic violence, broken family structures and cultural norms promoting early marriage, it provided grounds for sexual exploitation of the girls and concession of their fertility control decisions in an effort to get financial support. Adolescent mothers who were supported by their birth families through school continuation or an economic activity (with or without contraception use) avoided a repeat adolescent birth.

Our study results suggest the need to: alleviate household poverty and eradicate domestic violence; implement a school continuation policy or provide alternative forms of education for adolescent mothers; strengthen legislation against early marriage; and offer contraception services at each contact with the adolescent girls, including during postnatal care and childhood vaccination clinics which first-time adolescent mothers frequent. Beyond focusing on preventing index adolescent pregnancy, programs need to specifically place emphasis on preventing repeat adolescent pregnancy.

We suggest future studies explore appropriate approaches to advance economic empowerment of adolescent mothers, delivery of contraception services to vulnerable adolescents such as those in marriage, and implementation of a school continuation policy, in LMIC settings.

## Data Availability

The datasets used and/or analysed during the current study are available from the corresponding author on reasonable request.

## Abbreviations

IDI: Individual In-depth Interview
LMIC: Low- and Middle-Income Country
UDHS: Uganda Demographic and Health Survey
VHT: Village Health Team
